# Abdominal Abscess as a Rare Manifestation of Gout

**DOI:** 10.1177/10668969251318039

**Published:** 2025-02-20

**Authors:** Hanna Siatecka, Sara Faiz

**Affiliations:** 1 Department of Pathology and Immunology, 3989 Baylor College of Medicine, Houston, TX, USA

**Keywords:** gout, abdominal abscess

## Abstract

Gout is a common inflammatory arthritis in adults. Elevated levels of uric acid lead to the formation of monosodium urate crystals and their deposition in joints, and rarely, other parts of the body. The tissues most frequently involved are synovium, bone, cartilage, skin, tendon, ligament, and kidney. Here, we report an unusual presentation of gout manifesting as an abdominal abscess. A 60-year-old man with a history of opioid abuse, hypertension, and knee gouty arthritis presented to the emergency department with severe right knee pain. During hospitalization, he complained of pain in the left lower abdominal quadrant and developed coffee-ground emesis. Computed tomography showed pneumoperitoneum. The patient underwent laparotomy and was found to have gastric perforation and a large abdominal abscess. Drainage of the abscess revealed necrotic and hemorrhagic fragments of omentum. Microscopic examination showed adipose tissue with fat necrosis, acute inflammation, and fungal hyphae. Eosinophilic, amorphous deposits of polarizable needle-shaped crystals were observed in all sections, indicating the presence of monosodium urate crystals. The combined characteristics suggested intra-abdominal gout with concurrent fungal infection resulting from gastric perforation. This presentation is very rare. Only one occurrence of intra-abdominal gout mimicking a pelvic abscess has been reported in the literature.

## Introduction

The prevalence of gout among the US adult population is 3.9%.^1,2^ Gout is more common in men and is associated with obesity, hypertension, hyperlipidemia, diabetes mellitus, alcohol, chronic kidney disease, and diet. Pathogenesis is related to the elevation of serum uric acid which leads to monosodium urate crystal deposition in joints and other tissues. Following that, the inflammatory response occurs and generates acute symptoms.^3^ The crystals have a propensity to develop and deposit in avascular, low-temperature tissues, like joints, tendons, ligaments, or ears.^3,4^ Disease presents as a typical monoarticular flare, polyarticular flare, or as tophaceous gout. Most commonly (80%) one joint is affected, usually within the lower extremity including the first metatarsophalangeal and knee joints. It usually manifests with acute pain and is self-limiting.^5^ Other, not as frequent, sites of involvement include ankles, wrists, fingers, olecranon bursa, and very rarely hips, sternoclavicular joints and shoulders. The uncommon presentations described in literature include spine and sacroiliac joints.^6,7^

## Patient Presentation

**Figure 1.**
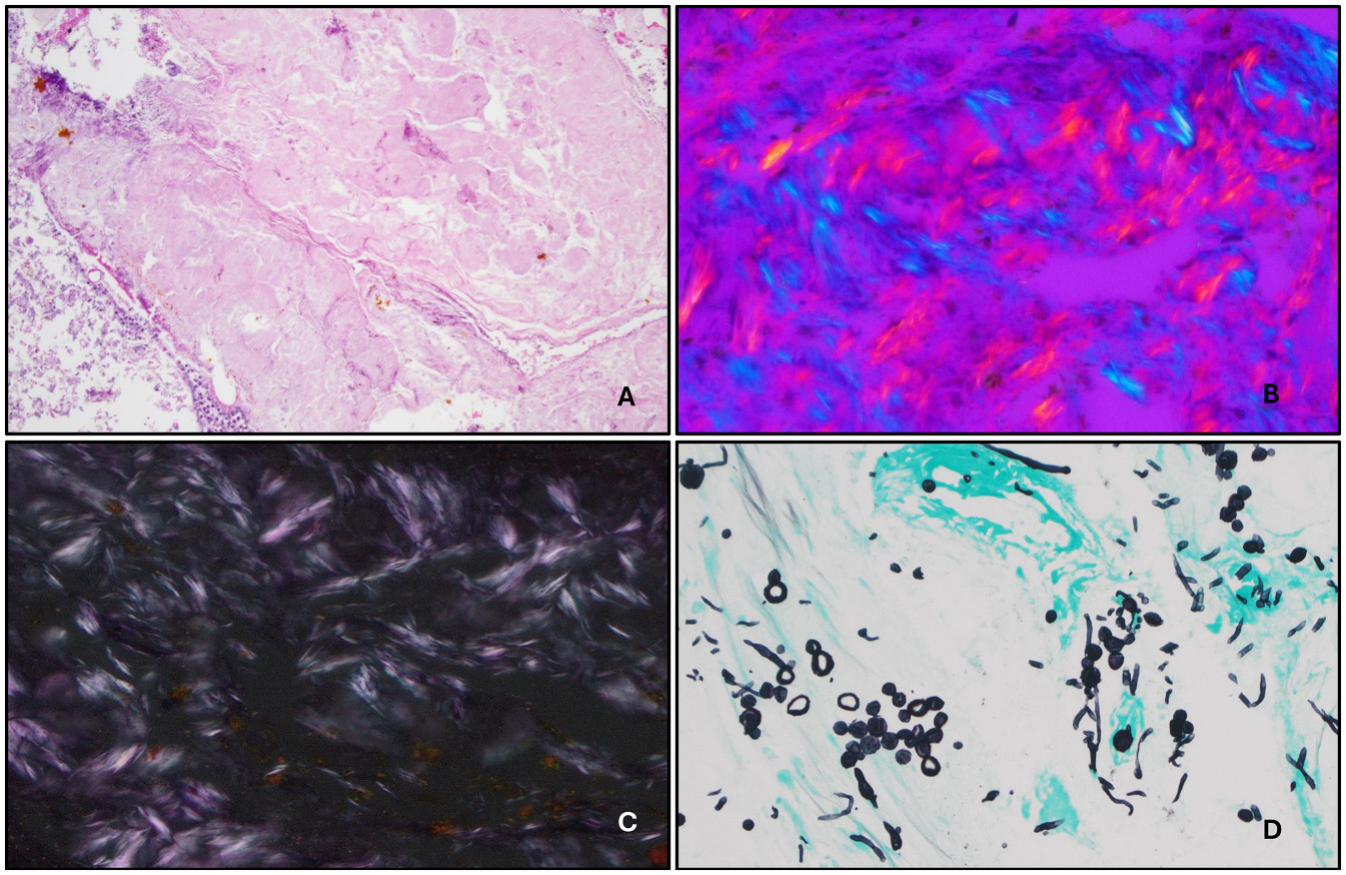
Omentum. (A) Gouty deposits with surrounding acute and chronic inflammation (100×). (B) Monosodium urate crystals under polarized light (400×). (C) Polarizable needle-shaped birefringent monosodium urate crystals (400×). (D) Fungal hyphae highlighted by positive GMS stain (400×).

A 60-year-old male patient with a history of opioid abuse, hypertension, untreated hepatitis C, and chronic right knee pain presented to the emergency department (ED) with a complaint of severe right knee pain. The patient was hospitalized in the ED multiple times during the past 6 months with the same complaint and always received naproxen for pain relief. A few months before, in the outside institution, right knee synovial fluid was sampled, and monosodium urate crystals were detected. Also, the right foot X-ray was performed and showed periarticular hyperdense foci adjacent to the first metatarsophalangeal and interphalangeal joints, which could reflect tophi related to gout. The serum uric acid concentration has never been checked and the treatments for gout were never administered. During the admission, he suddenly developed acute respiratory failure and acute kidney failure. Aspiration pneumonia was suspected. The blood cultures were drawn and showed gram-positive bacteremia. He also complained of left lower quadrant abdominal pain and developed coffee-ground emesis. Imaging studies confirmed a suspected perforation, and the patient underwent exploratory laparotomy. During the procedure, a large abdominal abscess was discovered and drained. The gastric ulcer was biopsied, and a microscopic exam revealed numerous fungal organisms in the superficial ulcerated layer. The Grocott-Gomori's methenamine silver (GMS) special stain was performed and identified fungal organisms which were most compatible with Candida species. The acid-fast bacilli (AFB) special stain was negative. The Warthin starry special stain did not identify Helicobacter pylori. The postoperative course has been complicated by percutaneous drainage of the intra-abdominal abscess, gastrointestinal bleeding from the ulcer, abdominal wall tissue necrosis requiring debridement and enteric leak. Following the drainage of the abscess, the fragment of the omentum was sent to the pathology lab and examined. The gross exam showed multiple tan-yellow fragments of hemorrhagic and necrotic omentum. The microscopic examination revealed adipose tissue with fat necrosis, acute inflammation, fungal hyphae, and green pigment, suggestive of bile. Eosinophilic, amorphous polarizable needle-shaped crystals were present in all sections, consistent with monosodium urate crystals (Figure 1A-C). The periodic acid-Schiff stain (PAS-fungus) and GMS stains (Figure 1D) were positive for fungal hyphae. The patient's clinical status slowly improved, following prolonged hospitalization due to wound healing.

## Discussion

Gout is a common inflammatory process resulting from the deposition of monosodium urate crystals in many tissues. Hyperuricemia results in deposition of crystals, subsequent inflammatory response, and tissue damage.^2^ There is increasing evidence that links gout to metabolic syndrome, a rising incidence of myocardial infarction and diabetes type 2, hence premature death.^1^ Musculoskeletal tissues are the most common locations for crystal deposition. Among those, bone, skin, tendon and ligament, spine, synovium, cartilage, and carpal tunnel are the most frequent sites.^2^ The non-musculoskeletal tissues or organs with gout described in the literature include the kidney, heart, eye, larynx, breast, intestine, lung, mesentery, prostate, urinary bladder, nose, middle ear, paranasal sinuses, nail, nerve, omentum, pancreas, and coronary arteries.^2,8^ The most frequently reported non-musculoskeletal sites are the heart, kidney, and eye.^2^

The microscopic examination of the tissue affected by gout shows crystal deposits as amorphous material surrounded by chronic inflammation. Giant cells are a common finding. The crystals are needle-shaped and have an amphophilic color. The microscopic slides should be examined under polarized light, where they are negatively birefringent and display either a yellow or blue color. If lined up parallel to light, they are yellow, and if aligned perpendicular to light they show blue (Figure 1B).^2^

Concurrent infections and gout are well documented in instances of septic arthritis. Diagnosing articular gout with a simultaneous infection, most commonly bacterial, can be challenging due to similar clinical features. Therefore, synovial fluid aspiration and microbiological culture are crucial to ensure an accurate diagnosis.^9^ Notably, however, there are no reported occurrences of visceral gout complicated by a fungal infection secondary to a perforated gastric ulcer.

The differential diagnosis for amorphous material within the abdominal cavity is broad, primarily encompassing complications related to perforations. Key considerations include pulse/hyaline ring granulomas, recently referred to as “seed storage cell granulomas,” and granulomatous reactions to foreign materials such as medications.^10,11^ Although rare, other possible causes include amyloidosis and gout.^12^

In pulse granulomas, the granuloma-inducing material, often derived from leguminous seeds, presents distinctively. The cells are typically large, ovoid, or round, featuring an outer cellulose-rich cell wall and granular cytoplasm. These granules, which vary in size, often stain positively with PAS, while the cell walls may exhibit birefringence under polarized light.^10^ Differentiating features from gout include the absence of needle-shaped crystals in pulse granulomas, which are characteristic of gout.

Certain medications, such as sodium polystyrene sulfonate (Kayexalate) and sevelamer, can lead to gastroenteritis, perforation, and intra-abdominal abscess formation, each with distinct crystalline morphology. Sodium polystyrene sulfonate forms bright purple crystals, while sevelamer forms yellow or pink crystals; both exhibit a fish-scale pattern. They can be further distinguished using the AFB stain: sodium polystyrene sulfonate appears black, and sevelamer appears magenta.^11,13,14^

Amyloidosis should also be considered in the context of amorphous material accumulation. This condition involves the deposition of misfolded proteins in tissues, typically with minimal stromal inflammation and a positive Congo red stain, unlike in gout, where crystals are present.^12^

Extraarticular gout should be always taken into consideration in a patient with a history of gouty arthritis. It is important to keep this differential in mind as a pathologist, because of differences in specimen handling. Proper triage would include submitting tissue in 100% alcohol; hence the formalin dissolves the urate crystals making the diagnosis challenging to make.^6^ In this patient, gout was an unexpected finding in the omentum, and the specimen was received in formalin. Despite this, the microscopic findings were characteristic and easily identifiable, confirming the diagnosis of longstanding, severe, and untreated gout.

## Conclusions

Abdominal abscess manifesting as gout is exceptionally uncommon. Despite the rarity, any mass-like lesions in patients with systemic gout should be considered in differential diagnosis. When clinical suspicion arises, it is essential to preserve the tissue specimen in a fresh state or an alcohol-based solution rather than in formalin. This precaution is necessary as formalin can dissolve the characteristic birefringent crystals observed under polarized light. In conclusion, gout is a common disease that can affect multiple organs and is not only seen in musculoskeletal tissues. It is an important cause of arthritis, however, its presence in other organs has been reported and should be considered.
